# Tin, Bismuth, and Tin–Bismuth Alloy Electrodeposition from Chlorometalate Salts in Deep Eutectic Solvents

**DOI:** 10.1002/open.201700045

**Published:** 2017-04-13

**Authors:** Luciana Vieira, Jennifer Burt, Peter W. Richardson, Daniel Schloffer, David Fuchs, Alwin Moser, Philip N. Bartlett, Gillian Reid, Bernhard Gollas

**Affiliations:** ^1^ Institute for Chemistry and Technology of MaterialsGraz University of TechnologyStremayrgasse 98010GrazAustria; ^2^ Chemistry, University of Southampton, HighfieldUniversity RoadSouthamptonSO17 1BJUK

**Keywords:** alloys, deep eutectic solvents, electrochemistry, electrodeposition, main group elements

## Abstract

The electrodeposition of tin, bismuth, and tin–bismuth alloys from Sn^II^ and Bi^III^ chlorometalate salts in the choline chloride/ethylene glycol (1:2 molar ratio) deep eutectic solvent was studied on glassy carbon and gold by cyclic voltammetry, rotating disc voltammetry, and chronoamperometry. The Sn^II^‐containing electrolyte showed one voltammetric redox process corresponding to Sn^II^/Sn^0^. The diffusion coefficient of [SnCl_3_]^−^, detected as the dominating species by Raman spectroscopy, was determined from Levich and Cottrell analyses. The Bi^III^‐containing electrolyte showed two voltammetric reduction processes, both attributed to Bi^III^/Bi^0^. Dimensionless current/time transients revealed that the electrodeposition of both Sn and Bi on glassy carbon proceeded by 3D‐progressive nucleation at a low overpotential and changed to instantaneous at higher overpotentials. The nucleation rate of Bi on glassy carbon was considerably smaller than that of Sn. Elemental Sn and Bi were electrodeposited on Au‐coated glass slides from their respective salt solutions, as were Sn–Bi alloys from a 2:1 Sn^II^/Bi^III^ solution. The biphasic Sn–Bi alloys changed from a Bi‐rich composition to a Sn‐rich composition by making the deposition potential more negative.

## 1 Introduction

Tin and its alloys are commonly used as lead‐free soft solder layers in the mass production of electronic components.^1^ Small amounts of Bi (≤4 wt %) are added to Sn solder to reduce whisker formation, and Sn alloys with higher Bi contents are soft solder candidates to replace tin–lead.^2^ Aqueous electroplating of tin, bismuth, and their alloys is usually performed from strongly acidic electrolytes based, for example, on methanesulfonic acid, because the respective salts are readily soluble and do not hydrolyze at low pH values.^1^, ^3^

Recently, the electrodeposition of p‐block elements, including bismuth and tin, from tetrabutylammonium chlorometalate salts of the elements in dichloromethane and in supercritical fluids was reported.^4^, ^5^ These reagents are crystalline solids, easy to handle, and not highly water or oxygen sensitive. Both tin and bismuth show straightforward redox behavior in these electrolytes. The surface tension of organic solvents such as dichloromethane and supercritical fluids is low and thus provide excellent wetting even for high aspect ratio recesses on nanopatterned substrates such as integrated circuits. However, these solvents are highly volatile and require a supporting electrolyte to achieve reasonable conductivity.

Deep eutectic solvents (DESs),^6^ on the other hand, although rather viscous, have very low volatility and possess good inherent conductivity and potential windows that are significantly larger than that of water. In contrast to most of the conventional room‐temperature ionic liquids, deep eutectic solvents are cheap and are considered truly green solvents, particularly if they are based on natural compounds such as choline chloride.^7^ Due to their good solubilization of metal salts and their insensitivity towards water and oxygen, deep eutectic solvents have been advocated as electrolytes for the electroplating of metals.^8^, ^9^

The electrodeposition of Sn from the type 1^10^ deep eutectic solvents AlCl_3_/1‐ethyl‐3‐methylimidazolium chloride^11^ and ZnCl_2_/1‐ethyl‐3‐methylimidazolium chloride^12^ was previously reported. For both studies, it was indicated that the speciation of Sn^II^ depended on the Lewis acidity of the electrolyte. Electrodeposition of tin from choline chloride based type 3 deep eutectic solvents containing either anhydrous SnCl_2_ 13, 14, 15 or SnCl_2_ **⋅**2 H_2_O^16^, ^17^, ^18^, ^19^ was also demonstrated. FAB‐MS indicated the presence of the trichlorostannate anion [SnCl_3_]^−^, but this method is not conclusive, because of possible fragmentation in the gas phase. Raman spectroscopy of strongly Lewis basic imidazolium chloride based ionic liquids showed [SnCl_3_]^−^ to be the dominating Sn^II^ species.^20^, ^21^

In contrast to tin, the electrochemistry of bismuth in deep eutectic solvents is less explored. The speciation of Bi^III^ in chloride‐containing aqueous^22^, ^23^, ^24^, ^25^, ^26^ and organic media^27^ as well as molten salts^28^, ^29^ has been studied. Whereas some uncertainty about the existence of pentachlorobismuthate(III) remains, the following complex equilibrium in chloride‐containing solutions [Eq. (1)] is generally accepted:^23^, ^27^

(1) $$\left[\mathrm{BiCl}{}_{4}\right]{}^{-}+2\;\mathrm{Cl}{}^{-}=\left[\mathrm{BiCl}{}_{6}\right]{}^{3-}$$

The electrochemistry of bismuth was previously studied in a Lewis acidic type 1 deep eutectic electrolyte of AlCl_3_/*N*‐(*n*‐butyl)pyridinium chloride containing 1 mm BiCl_3._ 30 In this medium, the cathodic reduction of Bi^III^ was shown to proceed via the low‐valence state intermediate Bi_5_^3+^. Furthermore, evidence for the formation of Bi^I[31]^ during the anodic oxidation of Bi was presented.^30^ The electrodeposition of bismuth from a 1:2 mixture of choline chloride/urea containing Bi_2_O_3_ was studied,^32^ and the electrodeposition of films of Bi, Te, and thermoelectric BiTe from choline chloride/malonic acid^33^ and from choline chloride/oxalic acid was also explored.^34^ Moreover, the electrodeposition of films of BiTeSe from choline chloride DESs with ethylene glycol, malonic acid, or oxalic acid^35^ and the electrodeposition of films of BiSe from choline chloride/malonic acid^36^ was demonstrated. The speciation of Bi^III^ in these DESs is unclear. Recently, the electrodeposition of Sn–Bi coatings on Cu from a DES comprising a 1:2 molar ratio of choline chloride/ethylene glycol (12CE) containing 0.05 m SnCl_2_, 0.05 m BiCl_3_, and 0.1 m H_3_BO_4_ as an additive was also reported,^37^ but the study focused more on the characterization of the resulting deposits than on the vaguely described electrochemistry.

Herein, we describe the electrodeposition of Sn, Bi, and their alloys from their corresponding tetrabutylammonium chlorometalate salts in 12CE and characterization of the metallic deposits.

## 2 Results and Discussion

### 2.1 Deposition of Elemental Sn and Bi

**Figure 1:**
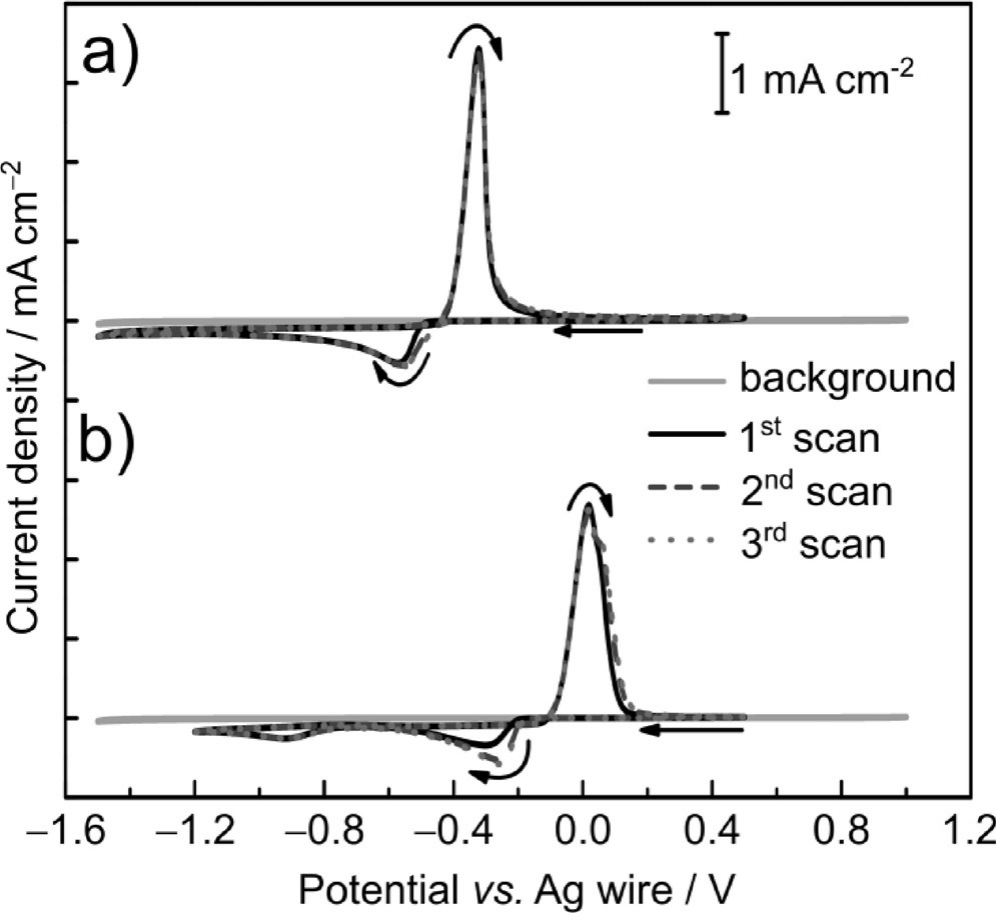
Cyclic voltammograms of a) 10 mmol L^−1^ [N*n*Bu_4_][SnCl_3_] and b) 10 mmol L^−1^ [N*n*Bu_4_][BiCl_4_] in 12CE on a GC disk electrode (0.07 cm^2^) at a scan rate of 50 mV s^−1^ at room temperature. The sweeps started at open‐circuit potentials of a)+0.20 and b)+0.23 V with a negative sweep direction. The light‐gray scans are the corresponding background CV curves (pure 12CE).

The cyclic voltammetry (CV) curves of 12CE solutions containing 10 mmol L^−1^ of [N*n*Bu_4_][SnCl_3_] and [N*n*Bu_4_][BiCl_4_] on a glassy carbon (GC) electrode are shown in Figure 1. From a comparison with the background CV curve, for which only the capacitive current is observed, it is clear that the cathodic and anodic peaks originate from the reduction of the metal ions in solution and the oxidation of the reduction products.

The CV curve in Figure 1 a shows a reduction peak potential (*E*_p_^red^) for Sn^II^ on GC at −0.571 V, followed by an oxidation peak potential (*E*_p_^ox^) at −0.322 V with a typical symmetrical shape for metal stripping in the anodic return sweep. At a potential of around +0.7 V, Sn^II^ species are irreversibly oxidized to Sn^IV^ (see Figure S1 in the Supporting Information). On the basis of the ratio of the anodic/cathodic charge, the coulombic efficiency for the Sn^II/0^ reduction of all three cycles is around 92 %, if complete anodic stripping of Sn is assumed. This value is considerably higher than the 70 % efficiency for the electrodeposition of tin from the same tin salt on gold in a CH_2_F_2_ supercritical fluid.^5^ Sn deposition on a GC electrode from a 1‐ethyl‐3‐methylimidazolium dicyanamide (EMIm‐DCA) ionic liquid gave a coulombic efficiency of 40 %.^38^

As for the reduction of Bi^III^, two reduction peaks are observed at −0.300 and −0.923 V, and this is followed by a single oxidation/stripping peak at +0.019 V (Figure 1 b). Because only one oxidation peak is observed, it is unlikely that two different kinds of deposits are formed. The total anodic charge over the total cathodic charge (both reduction peaks) is around 97 % for all three cycles, which indicates that most of the Bi is anodically stripped and that no or very minor irreversible side reactions occur in this potential range. This suggests that bismuth is deposited from two different bismuth species, which is supported by the coulombic efficiencies calculated from the total anodic over cathodic charges in the CV curves with different cathodic switching potentials (see Figure S2). The ratio of anodic over cathodic charge is around 100(±2) %, irrespective of whether only the first or both reduction processes are included.

Whereas blocking of the glassy carbon surface by an adsorbed layer of choline ions was reported to play a role in the electrodeposition of zinc,^39^, ^40^, ^41^ the reduction potentials for Sn^II^ and Bi^III^ are significantly more positive than those reported for surface blocking (≈−2.0 V and below). Hence, blocking effects such as those reported during the electrodeposition of zinc from the same electrolyte can be dismissed in the potential range of −1.5 to +1.0 V versus Ag wire used here for Sn and Bi.

**Figure 2:**
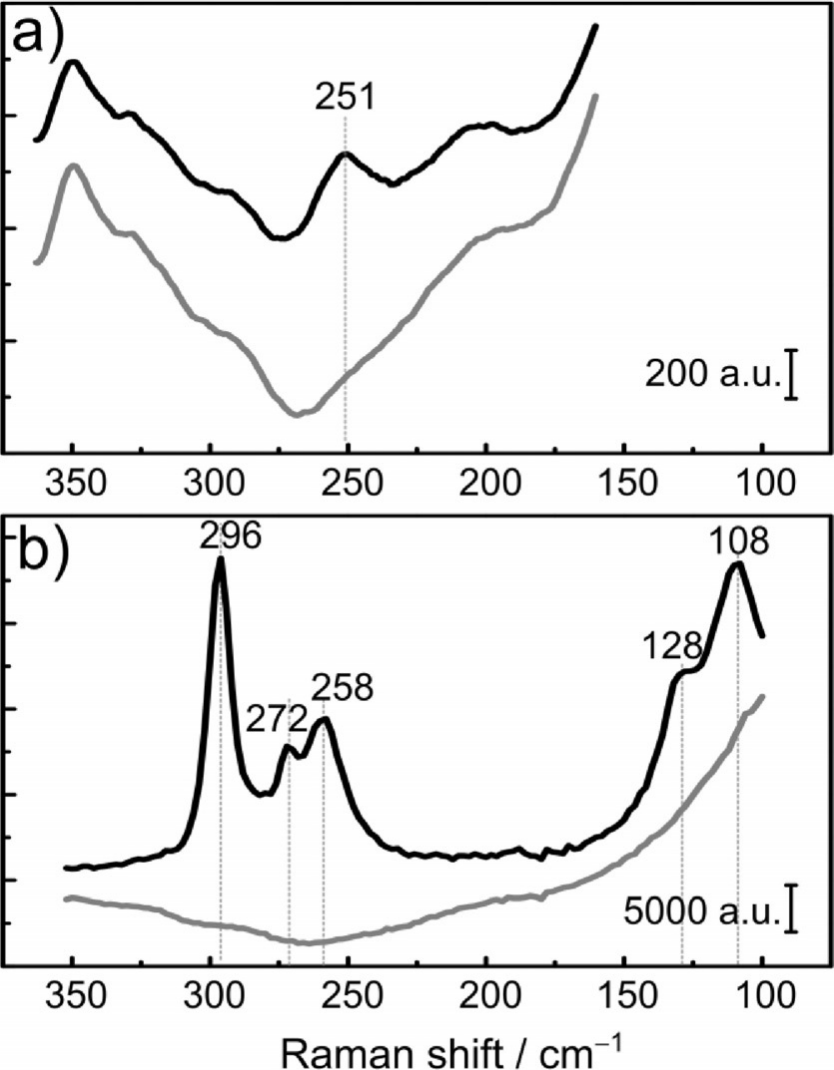
Raman spectra of the 12CE electrolyte containing a) 10 mmol L^−1^ [N*n*Bu_4_][BiCl_4_]. and b) 10 mmol L^−1^ [N*n*Bu_4_][SnCl_3_]. The background spectra of neat 12CE are shown in light gray.

The speciation of the chlorometalates in the 12CE electrolyte was investigated by Raman spectroscopy. The Raman spectra show bands corresponding to vibrations of Bi−Cl (Figure 2 a) and Sn−Cl (Figure 2 b).

The Raman spectrum of 12CE containing [N*n*Bu_4_][BiCl_4_] exhibits a band at a Raman shift of 251 cm^−1^. This stretching vibration appears at a wavenumber similar to those reported for [BiCl_6_]^3−^ in aqueous^24^, ^25^ and molten salts solutions,^28^, ^29^ as well as for isolated crystals of [C_6_H_18_N_3_][BiCl_6_]_._ 42 Bands related to other bismuth species are not observed. This suggests that only one bismuth species is present in the bulk solution and that the second reduction peak in the CV curves originates from an intermediate formed upon the first reduction. However, it is important to consider that the concentration of [N*n*Bu_4_][BiCl_4_] in the electrolyte is quite low (10 mmol L^−1^) and the band observed is weak. Using higher Bi^III^ concentrations is constrained by the low solubility of the salt. On the basis of the Raman data alone, the presence of a second bismuth species can thus not be completely ruled out.

The relative reduction potentials of the chlorometalates determined for Bi/Bi^III^ (−0.079 V) and Sn/Sn^II^ (−0.419 V) in 12CE in this work compare well with those measured in CH_2_Cl_2_ solution (−0.13 and −0.55 V, respectively)^4^ and in supercritical CH_2_F_2_ (−0.3 and −0.7 V, respectively),^5^ each containing an excess amount of [N*n*Bu_4_]Cl as the supporting electrolyte. On the basis of the Raman data and by comparison of the relative reduction potentials, it seems most likely that the first reduction peak (*E*_p_^red^=−0.30 V) is associated with reduction of the more dominant (≈90 %) [BiCl_6_]^3−^ anion. The second reduction (−0.92 V) also produces elemental Bi. However, the nature of the bismuth species giving rise to this reduction wave is not easy to establish given the propensity of bismuth(III) ions to form halide‐bridged dimers and higher oligomers and the variable (high) coordination numbers that Bi^III^ may adopt.^43^ The [Bi_2_Cl_11_]^5−^ and [Bi_2_Cl_10_]^4−^ dimers are likely candidates, because they bear higher negative charges and have a chloride/bismuth ratio similar to that of [BiCl_6_]^3−^. Also, the possibility of ethylene glycol taking part in the coordination^44^ cannot be ruled out.

Illner et al.^20^ studied the speciation of tin anions by ^119^Sn NMR and ^1^H NMR spectroscopy in 1‐butyl‐3‐methylimidazolium chloride (C_4_mimCl) solutions containing *cis*‐[Pt(PPh_3_)_2_Cl_2_] and different molar fractions of SnCl_2_ (*χ* ${}_{\mathrm{SnCl}{}_{2}}$ ). The authors observed a decrease in the ^119^Sn chemical shift upon decreasing *χ* ${}_{\mathrm{SnCl}{}_{2}}$ and suggested the formation of [SnCl_4_]^2−^ anions in Lewis basic media. At *χ* ${}_{\mathrm{SnCl}{}_{2}}$ <0.5, the NMR spectra only showed a single ^119^Sn resonance, which indicated that only a single chlorostannate species was present in the Lewis basic solution.

The anionic speciation of chlorostannates(II) in ionic liquids was systematically investigated by Currie et al.^21^ X‐ray photoelectron spectroscopy, ^119^Sn NMR spectroscopy, Raman spectroscopy, and differential scanning calorimetric studies of different molar fractions of SnCl_2_ in 1‐octyl‐3‐methylimidazolium (C_8_mim) and 1‐ethyl‐3‐methylimidazolium (C_2_mim) solutions showed Cl^−^ and [SnCl_3_]^−^ to be the only significant anions present in the Lewis basic medium. It was concluded that no [SnCl_4_]^2−^ was present under these conditions.

Fast atom bombardment (FAB) mass spectrometry, reported by Abbott's group, suggested the formation of [SnCl_3_]^−^ in 12CE electrolyte.^13^, ^45^ However, as FAB detects only the most stable species in the gas phase, the results were not conclusive with respect to speciation in solution. ^119^Sn NMR spectroscopy of a 10 mmol L^−1^ solution of [N*n*Bu_4_][SnCl_3_] in 12CE (Figure S4) revealed a sharp resonance at *δ*=−160.7 ppm. This value is similar to the chemical shifts previously reported for Lewis basic imidazolium‐based ionic liquids^20^, ^21^ and contrasts the singlet observed at *δ*=−40.8 ppm for [N*n*Bu_4_][SnCl_3_] in CH_2_Cl_2_ solution, which remains unchanged in the presence of a tenfold excess amount of [N*n*Bu4]Cl.^5^ However, as NMR spectroscopy has a slow experimental timescale, the exchange between chloroanions may give only an averaged chemical shift.

The Raman spectrum of [N*n*Bu_4_][SnCl_3_] in 12CE shows five Sn−Cl stretching vibrations at Raman shifts between 300 and 100 cm^−1^, and this is consistent with that reported by Currie et al.^21^ for [SnCl_3_]^−^ in Lewis basic C_2_mimCl–SnCl_2_ solutions (*χ* ${}_{\mathrm{SnCl}{}_{2}}$ =0.2). For a *C*_3*v*_ symmetric species, group theory predicts four Raman bands, two stretching modes (a_1+_e) and two bending modes (a_1+_e), and these occur at Raman shifts of 297, 256, 128, and 103 cm^−1^ for [SnCl_3_]^−^ in diethyl ether solution.^46^ The bands shown here for the 12CE solution are at higher wavenumbers than those reported for [SnCl_3_]^−^ in imidazolium chloride ionic liquids.^21^ This blueshift indicates a change in the chemical interaction of the [SnCl_3_]^−^ species for a higher‐energy system. As the 12CE electrolyte has a high concentration of chloride ions (4.5 mol L^−1^), the molar fraction of SnCl_2_ in this DES is 0.002 (for a 10 mmol L^−1^ solution), which is 200‐fold lower than that of the reported Lewis basic imidazolium system.^21^

**Figure 3:**
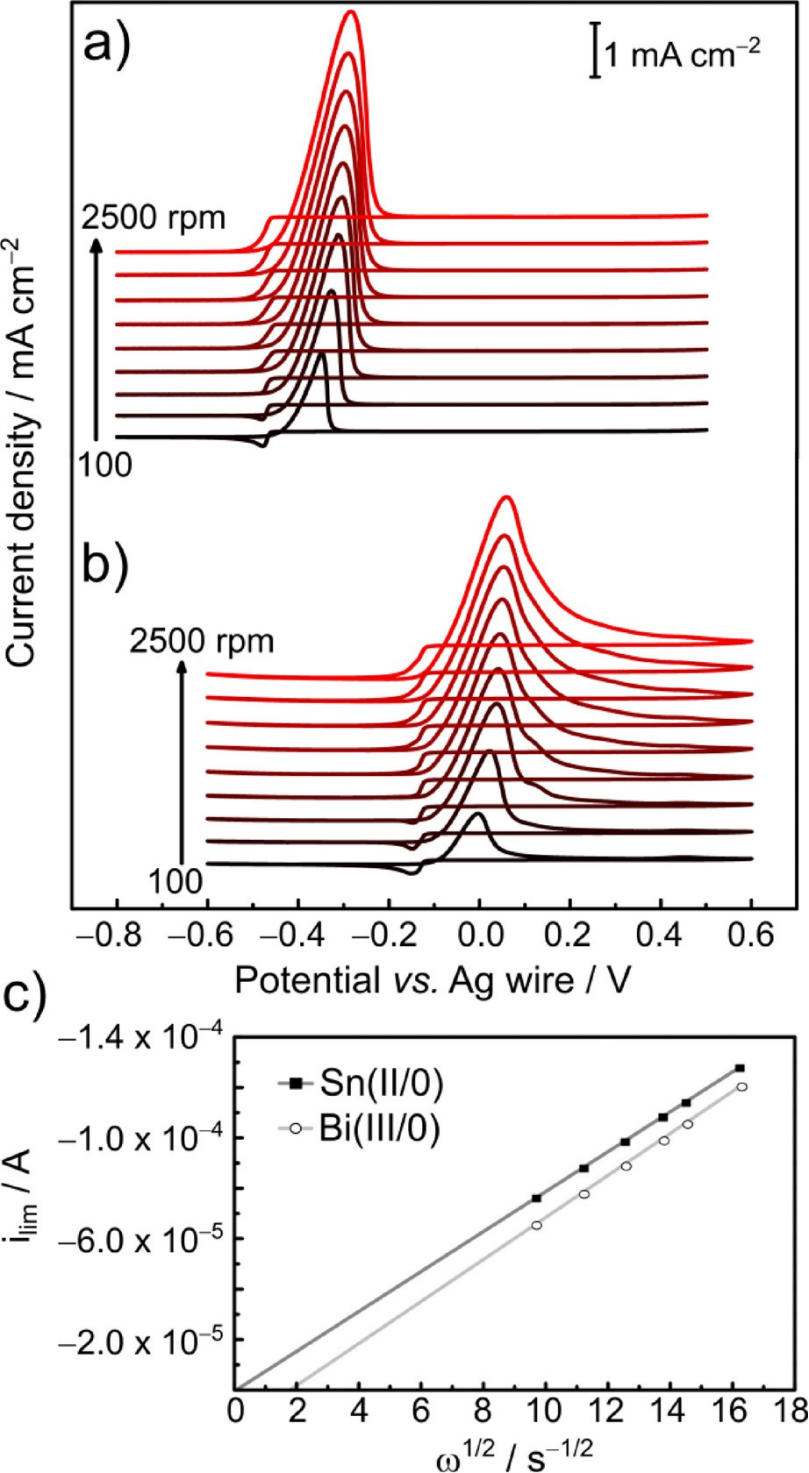
RDE CV curves of 12CE containing a) 10 mmol L^−1^ [N*n*Bu_4_][SnCl_3_] and b) 10 mmol L^−1^ [N*n*Bu_4_][BiCl_4_] on a GC disk electrode (0.126 cm^2^) at a scan rate of 5 mV s^−1^ and rotational rates of 100, 300, 600, 900, 1200, 1500, 1800, 2000, and 2500 rpm. c) Corresponding Levich plot for rotational rates ≥900 rpm and limiting currents collected at −0.6 and −0.3 V for Sn^II^ and Bi^III^ solutions, respectively.

A set of rotating disk electrode (RDE) voltammograms was measured on a glassy carbon electrode at rotational rates ranging from 100 to 2500 rpm (Figure 3). The RDE CV curves show an increase in the current density proportional to the rotation rate for both metal ions. At a scan rate of 5 mV s^−1^, well‐defined limiting currents are obtained at rotational rates higher than 900 rpm for both Bi^III^‐ and Sn^II^‐containing electrolytes. In the electrolyte containing [N*n*Bu_4_][BiCl_4_], a second steady‐state reduction wave (≈10 % of the height of the main wave, see Figure S5) remains at a potential of −0.92 V at scan rates smaller than 10 mV s^−1^, and this is consistent with the presence of two Bi species in equilibrium in the solution.

The diffusion coefficients (*D*) of the tin and bismuth electroactive species were calculated with the Levich equation^47^ from the slopes shown in Figure 3 c by assuming bulk concentrations of 10 mm for both [SnCl_3_]^−^ and [BiCl_6_]^3−^. The diffusion coefficient for [SnCl_3_]^−^ was found to be 1.49×10^−7^ cm^2^ s^−1^ at −0.6 V, and the diffusion coefficient for [BiCl_6_]^3−^ was found to be 8.74×10^−8^ cm^2^ s^−1^ at −0.3 V. Both Levich plots show straight lines with correlation coefficients higher than 0.99. In contrast to the regression line for [SnCl_3_]^−^ reduction, the line for [BiCl_6_]^3−^ does not cross the origin. This is probably a consequence of the complex equilibrium of the Bi species in solution. The value of 8.74×10^−8^ cm^2^ s^−1^ for the diffusion coefficient of [BiCl_6_]^3−^ should thus be considered a minimum value.

The diffusion coefficient for Bi^III^ in 1.0 m HNO_3_ aqueous electrolyte was found to be 1.7×10^−6^ cm^2^ s^−1^, as calculated from a Randles–Sevcik plot.^48^ The diffusion coefficient for Bi^III^ in 0.5 m LiNO_3_/ethylene glycol at 50 °C was determined to be 1.3×10^−6^ cm^2^ s^−1^ from RDE measurements.^49^ Given that ionic liquids have higher viscosity than aqueous solutions, the diffusion coefficient is usually smaller in such melts. In a 2:1 AlCl_3_/*N*‐(*n*‐butyl)pyridinium chloride ionic liquid, the diffusion coefficient of the electroactive bismuth species, namely, Bi_5_^3+^ ions, calculated from the Levich equation, was found to be 2.0×10^−7^ cm^2^ s^−1^,^30^ which is significantly larger than that found here in 12CE.

The diffusion coefficient of 1.49×10^−7^ cm^2^ s^−1^ calculated for [SnCl_3_]^−^ is similar to the value of 1.96×10^−7^ cm^2^ s^−1^ reported for SnCl_2_ **⋅**2 H_2_O dissolved in 12CE.^18^

**Figure 4:**
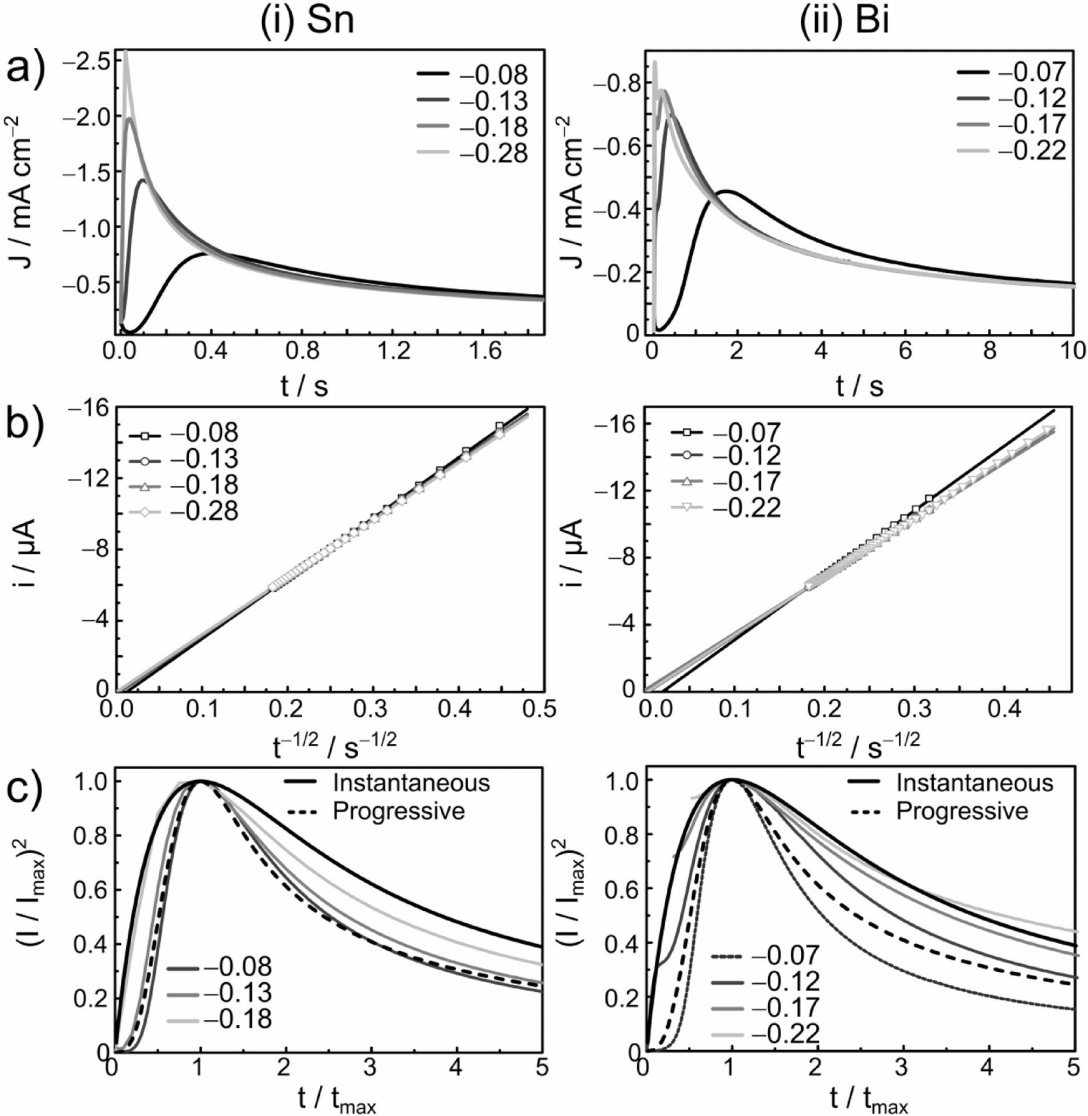
a) Chronoamperograms of 12CE containing i) 10 mmol L^−1^ [N*n*Bu_4_][SnCl_3_] and ii) 10 mmol L^−1^ [N*n*Bu_4_][BiCl_4_] on a 0.07 cm^2^ glassy carbon disk electrode, b) Cottrell plots, and c) dimensionless experimental and theoretical current–time transients for 3D instantaneous and progressive nucleation. The potential was stepped from +0.2 V (60 s) to the indicated overpotential (30 s).

Chronoamperometric experiments were performed for both electrolytes (Figure 4) by stepping the potential from a value at which no faradaic reactions occurred (+0.2 V) to overpotentials sufficiently negative to initiate nucleation and growth on a GC electrode. These overpotentials were chosen on the basis of the peak potentials in the CV curves presented in Figure 1 with respect to the equilibrium potentials, for which the reverse trace crosses the zero current axis.

Typical nucleation peaks are observed in the current–time transients for Sn^II^ and Bi^III^. After the capacitive decay, the current increases to a maximum (*I*_max_) at a certain time (*t*_max_). It is notable that the nucleation of Sn is much faster than that of Bi on the GC electrode, that is, the values of *t*_max_ are much shorter for the transients of [SnCl_3_]^−^ reduction.

The Cottrell plots shown in Figure 4 b exhibit good linear correlation for the last seconds of the measurement, during which time the current is diffusion controlled. Diffusion coefficients calculated by means of the Cottrell equation^47^ are shown in Table 1 for both electrolytes. The *D* values calculated from chronoamperometry are in good agreement with those calculated from the Levich equation, although the former data are more reliable for cases in which nucleation is involved. Also here, the values found for [BiCl_6_]^3−^ are minimum values, because of its equilibrium with a second species.

**Table 1:** Diffusion coefficients calculated from the Levich plots in Figure 3 c and the Cottrell plots in Figure 4 b for the tin and bismuth chlorometalate salts in 12CE.

Equation	*E* [V vs. Ag wire]	*D* [cm^2^ s^−1^]	*E* [V vs. Ag wire]	*D* [cm^2^ s^−1^]
	[N*n*Bu_4_][SnCl_3_]		[N*n*Bu_4_][BiCl_4_]	
Levich	−0.60	1.49×10^−7^	−0.30	8.74×10^−8^
Cottrell	−0.50	1.52×10^−7^	−0.15	7.29×10^−8^
	−0.55	1.42×10^−7^	−0.20	6.93×10^−8^
	−0.60	1.39×10^−7^	−0.25	6.93×10^−8^
	−0.70	1.36×10^−7^	−0.30	7.00×10^−8^

Dimensionless experimental transients were plotted together with the theoretical curves of the limiting cases for 3D instantaneous and progressive nucleation according to the Sharifker–Hills equations.^50^, ^51^, ^52^ In [SnCl_3_]^−^‐containing electrolytes, the nucleation at overpotentials of −0.08 and −0.13 V resembles the progressive model. Applying more negative overpotentials of −0.18 and −0.28 V changes the nucleation mechanism to instantaneous, which is consistent with the reports for SnCl_2_ in 12CE electrolyte at −1.20 V versus SCE (standard calomel electrode saturated with choline chloride) and 75 °C on GC.^52^ Similarly, the nucleation of tin on GC was shown to be instantaneous in 44.4–55.6 mol % AlCl_3_/EMImCl^11^ and 25–75 mol % EMImCl/ZnCl_2_ melts.^12^ On the other hand, progressive nucleation was found for Sn deposition from 1‐butyl‐1‐methylpyrrolidinium bis(trifluoromethylsulfonyl)imide (BMP‐TFSI) on Pt electrodes,^53^ whereas for the EMIm‐DCA ionic liquid, the experimental dimensionless plot fell between the two limiting cases on a GC electrode.^38^

DES solutions containing Bi^III^ resemble progressive nucleation at overpotentials of −0.07 and −0.12 V, whereas at more negative overpotentials of −0.17 and −0.22 V, the dimensionless transients fit the instantaneous nucleation model. According to the Sharifker–Hills models for 3D nucleation with hemispherical‐diffusion‐controlled growth,^52^ the nuclei form immediately at the beginning of the potential step for the instantaneous case, whereas for the progressive model, the nucleation sites are gradually activated with time. For both chlorometalate systems studied, the nucleation changes from progressive to instantaneous upon increasing the overpotential.

### 2.2 Sn and Bi Codeposition

**Figure 5:**
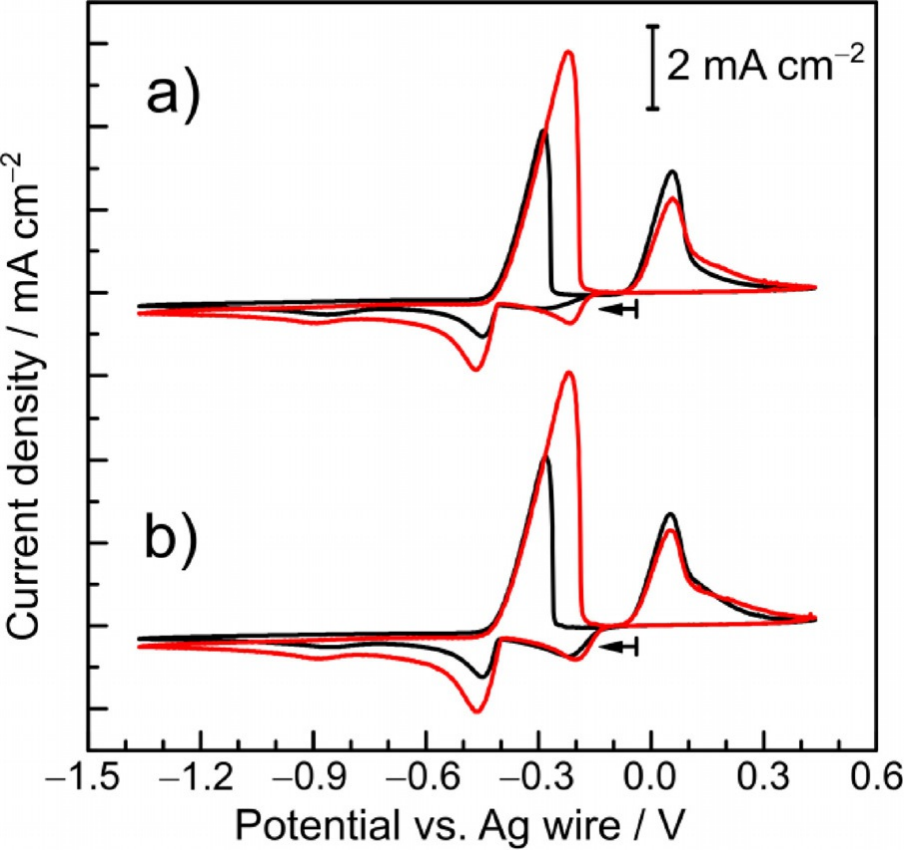
a) First voltammetric cycles at a GC disk electrode (0.126 cm^2^) of 12CE containing [NBu_4_][SnCl_3_]/[NBu_4_][BiCl_4_] at a concentration ratio of 1:1 (10/10 mmol L^−1^, black curve) and 2:1 (20/10 mmol L^−1^, red curve). The CV curves started in the negative direction from the open‐circuit potential of −0.03 V with a scan rate of 50 mV s^−1^. b) Second consecutive cycles of the CV curves in Figure 5 a.

According to the binary phase diagram of Sn and Bi,^54^ both metals have very low mutual solubility. Unlike the Sn–Cu system,^55^ they do not form solid solutions or intermetallics but instead a two‐phase mixture of both components. Figure 5 a shows the first CV cycles for 12CE solutions containing different concentrations of [NBu_4_][SnCl_3_] and [NBu_4_][BiCl_4_] at Sn^II^/Bi^III^ molar ratios of 1:1 (black curve) and 2:1 (red curve).

Both CV cycles show three well‐defined reduction peaks and two oxidation peaks. In the 1:1 ratio electrolyte, the reduction of Bi^III^ occurs at a potential of −0.29 V, and this is followed by a second reduction peak at a potential of −0.86 V. The reduction of Sn^II^ lies in between the two bismuth reduction peaks, at a potential of −0.44 V. In the anodic sweep, the dissolution of Sn takes place with a peak potential of −0.29 V, and this is followed by the dissolution of Bi more positively at a peak potential of +0.06 V.

Although the concentration of Bi^III^ in both electrolytes is the same, the first reduction peak of Bi^III^ in the 1:1 ratio electrolyte is significantly broader and has a smaller peak current than that in the 2:1 ratio electrolyte. In the second consecutive cycles shown in Figure 5 b, this difference almost disappears. Chronoamperometry already demonstrated (see above) that nucleation of Bi on glassy carbon was much slower than that of Sn. This is the reason for the drawn‐out peak of the first Bi^III^ reduction in Figure 5 a, and this can also be observed in the first cycle of Figure 1 b. Table 2 lists the cathodic and anodic charges consumed during the respective first and second cycles in Figure 5. It is clear that up to 10 % of the metal deposited during the first cycle remains on the electrode. Consequently, the effect of slow Bi nucleation is almost absent during the second cycle in both electrolytes. Given that the ratio of the anodic charge to the cathodic charge in all cycles was at least 90 % and that not all of the deposited metal was anodically stripped, the coulombic efficiencies of the electrodepositions on these timescales are probably all close to 100 %. The double‐layer structure in the 2:1 electrolyte seems to differ somewhat from that in the 1:1 electrolyte and allows for faster nucleation of Bi already during the first cycle.

**Table 2:** Cathodic (*Q*_c_) and anodic charges (*Q*_a_) consumed during CV (Figure 5) for the tin and bismuth chlorometalate electrolytes.

Cycle	*Q*_c_ [mC]	*Q*_a_ [mC]	*Q*_a_/*Q*_c_	*Q*_c_ [mC]	*Q*_a_ [mC]	*Q*_a_/*Q*_c_
	Sn^II^/Bi^III^ (1:1)			Sn^II^/Bi^III^ (2:1)		
1	1.048	0.957	0.91	1.633	1.472	0.90
2	1.156	1.069	0.92	1.769	1.632	0.92

### 2.3 Deposit Characterization

Sn, Bi, and Sn–Bi coatings were deposited potentiostatically on gold‐sputtered glass substrates at different potentials. Sn–Bi deposits were prepared from a 2:1 Sn^II^/Bi^III^ solution. The CV curves of Sn^II^, Bi^III^, and Sn^II^/Bi^III^ (2:1 molar ratio) in 12CE on gold electrodes can be found in the Supporting Information. The shift of the main stripping peak in the case of Sn to more positive potentials relative to the position of the main stripping peak in the CV curves on GC indicates the formation of intermetallic phases between Sn and Au (see below).

**Figure 6:**
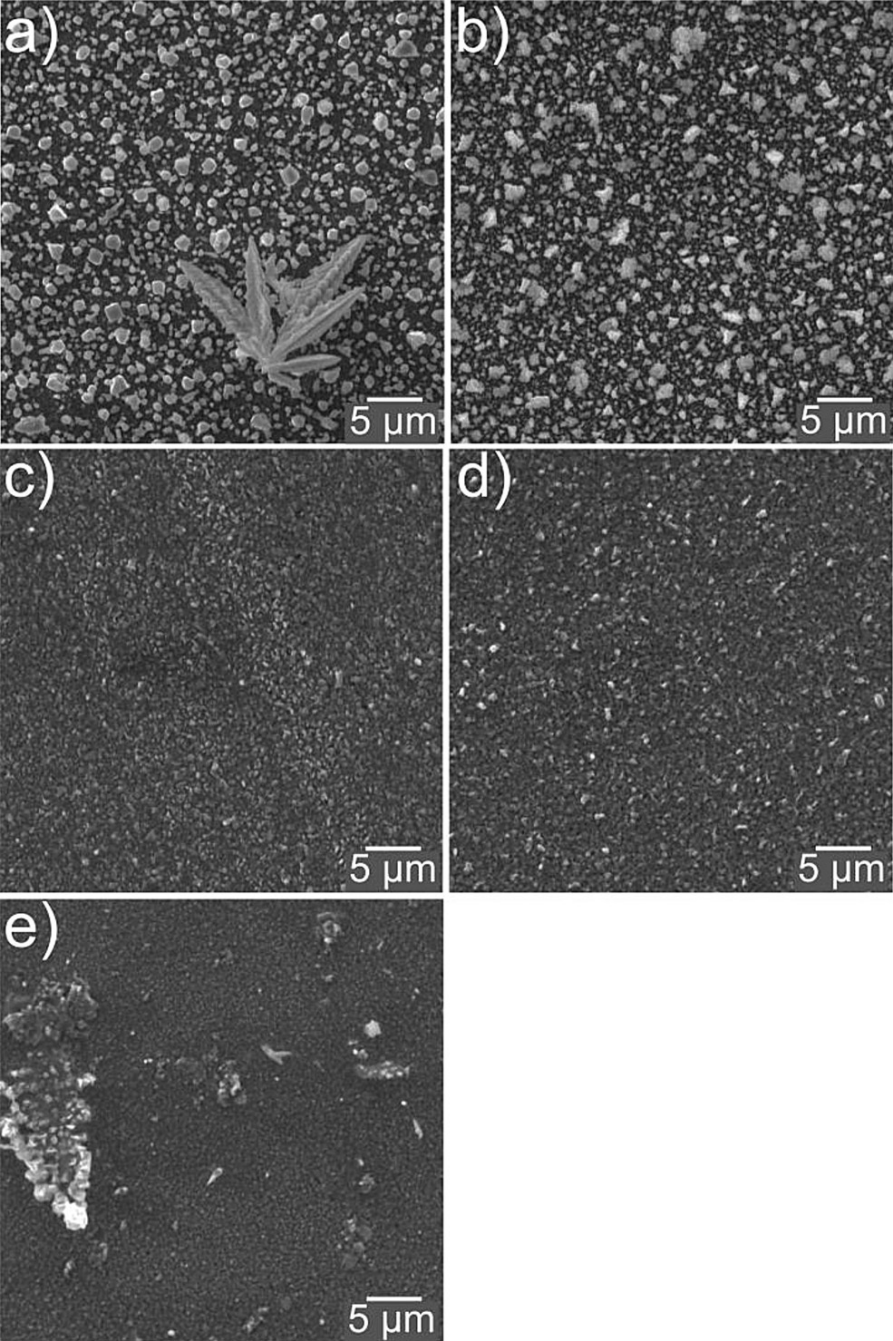
SEM images of a) Sn and b) Bi deposited from 10 mm chlorometalates in 12CE at −0.6 and −0.25 V, respectively, and of Sn–Bi alloys deposited from 20 mm Sn^II^/10 mm Bi^III^ chlorometalates in 12CE at c) −0.4 V, d) −0.5 V, and e) −0.6 V. All samples were electrodeposited at room temperature on gold‐sputtered glass slides. Sn and Bi coatings were deposited for 106 and 53 min, respectively, until a nominal coating thickness of 1 μm was reached. Sn–Bi samples were deposited for 30 min.

Top‐view scanning electron microscopy (SEM) images (Figure 6) show that elemental Sn and Bi deposited at −0.6 and −0.25 V, respectively, have a coarse‐grained morphology with the presence of some dendrites on the Sn deposits. Sn–Bi coatings were deposited at three different potentials of −0.4, −0.5, and −0.6 V. It was expected that the Sn/Bi ratio would increase by making the deposition potential more negative, because the reduction potential of Sn^II^ is more negative than that of Bi^III^. Energy‐dispersive X‐ray (EDX) spectroscopy analyses (Figure S3) revealed Sn/Bi ratios of 35:65, 53:47, and 59:41 wt % for samples deposited at −0.4, −0.5, and −0.6 V, respectively. Codeposition at the more‐negative potential resulted in an excess amount of Sn in the deposits, which is consistent with the presence of dendrites in this sample (Figure 6 e).

The bismuth deposits from supercritical fluids showed a dendritic morphology on both the TiN and glassy carbon substrates,^4^ whereas bismuth deposited on gold was reported to be quite smooth.^5^ Needlelike structures were observed by atomic‐force microscopy (AFM) analysis of Bi deposits from acidic chloroaluminate ionic liquids^56^ and from aqueous acidic solutions.^57^ Sn deposition from [N*n*Bu_4_][SnCl_3_] in supercritical difluoromethane led to flowerlike morphologies.^5^ Yet, Sn dendrites were also obtained from SnCl_2_ **⋅**2 H_2_O in the same deep eutectic electrolyte used here^16^ as well as from SnCl_2_ in the choline chloride/urea (1:2 molar ratio) DES.^13^

**Figure 7:**
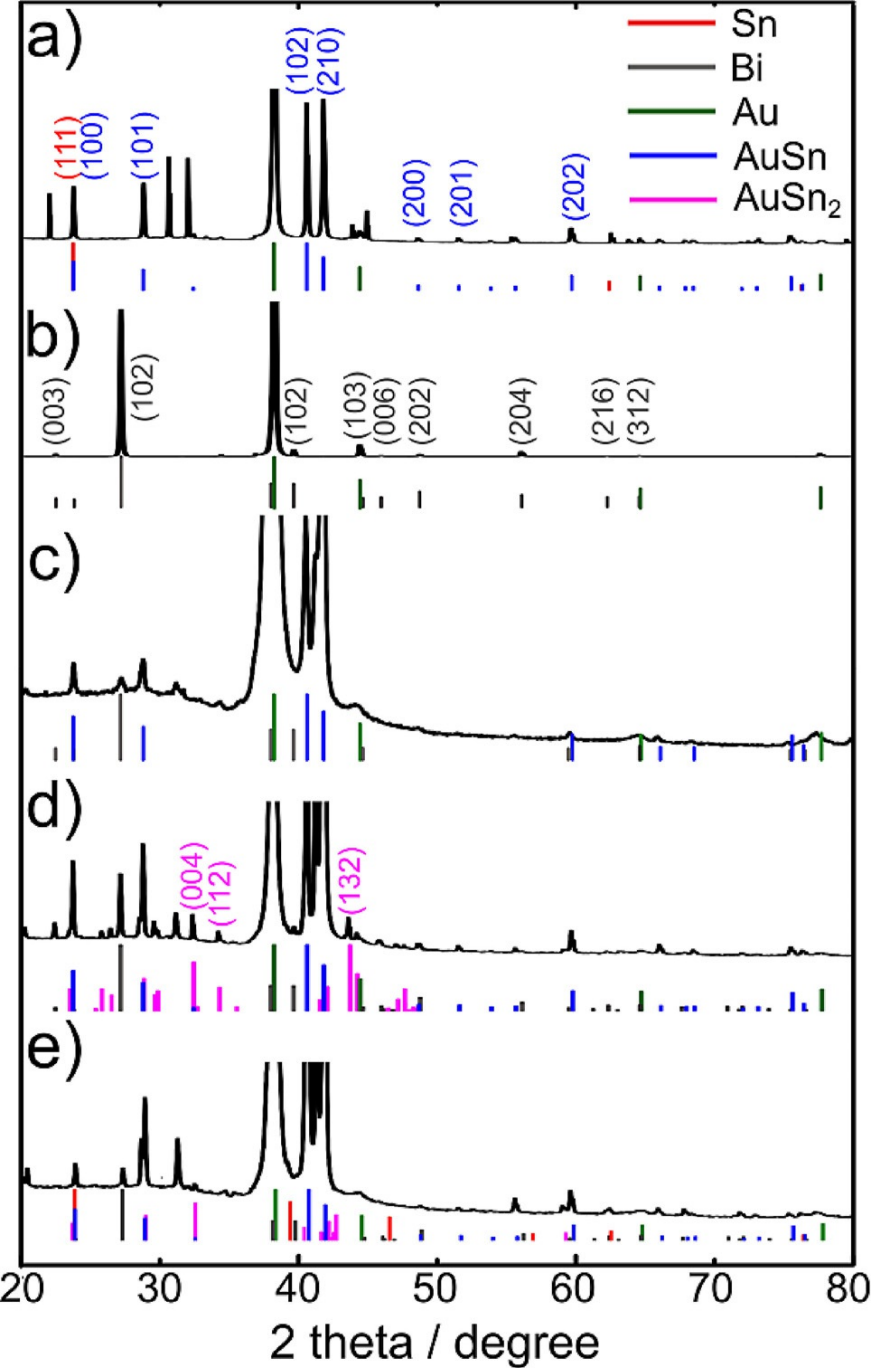
XRD patterns of a) Sn (−0.6 V) and b) Bi (−0.25 V) deposited from 10 mm chlorometalates in 12CE as well as Sn–Bi coatings deposited from 20 mm Sn^2+^/10 mm Bi^3+^ chlorometalates in 12CE at c) −0.4 V, d) −0.5 V, and e) −0.6 V on gold‐sputtered glass slides.

Different crystalline phases were identified by analyzing the coatings by X‐ray diffraction, as presented in Figure 7. Besides bismuth (ICSD 53797), tin (ICSD 70128), and the gold substrate (ICSD 52700), reflections corresponding to the intermetallic AuSn (ICSD 56262) and AuSn_2_ (ICSD 415968) phases are observed.

Studies of tin electrodeposited on gold electrodes from supercritical difluoromethane^5^ as well as from imidazolium‐11, 58 and pyrrolidinium‐based ionic liquids^59^, ^60^ also revealed the formation of Au–Sn intermetallics. Such surface alloys were detected after deposition of as little as one monolayer of tin.^11^ The latter can form several binary phases with gold, including AuSn, AuSn_2_, and Au_3_Sn.^54^

The diffraction pattern of the electrodeposited bismuth resembles those of bismuth coatings deposited on Au from supercritical difluoromethane^5^ and on TiN from [N*n*Bu_4_][BiCl_4_] in dichloromethane.^4^

## 3 Conclusions

We described the electrodeposition of Sn, Bi, and binary alloys of the two metals from tetrabutylammonium chlorometalate salts in a choline chloride/ethylene glycol (1:2) deep eutectic solvent (DES) with high coulombic efficiency. The Raman spectra of the deep eutectic solvent containing Sn^II^ salts showed that the dominant species in the DES was [SnCl_3_]^−^, with a Sn^II/0^ redox potential of −0.42 V versus Ag wire. In the case of Bi^III^, the speciation was not so clear, but the reduction potentials and Raman data pointed to [BiCl_6_]^3−^ as the dominant ion, with a Bi^III/0^ redox potential of −0.08 V versus Ag wire. This hexachlorobismuthate(III) was found to be in equilibrium with a second species, the structure of which remains unclear, but it could also be reduced to the metal at a reduction peak potential of about −0.9 V.

The diffusion coefficients of [SnCl_3_]^−^ and [BiCl_6_]^3−^ were calculated from Levich and Cottrell equations. The values from both methods are in good agreement and revealed that the diffusion coefficient of [SnCl_3_]^−^ was 1.4×10^−7^ cm^2^ s^−1^, whereas that of [BiCl_6_]^3−^ was only 7×10^−8^ cm^2^ s^−1^. The latter has to be considered a minimum value, because the reduction of the Bi^III^ species involves a complex equilibrium.

The chronoamperometric behavior indicated that the electrodeposition of both Sn and Bi on glassy carbon proceeded through 3D‐progressive nucleation at low overpotentials and changed to instantaneous nucleation at higher overpotentials. The nucleation of Bi on glassy carbon was considerably slower than that of Sn.

Scanning electron microscopy/energy‐dispersive X‐ray spectroscopy analysis of the Sn, Bi, and Sn–Bi deposits showed that increasing the Sn/Bi weight ratio from 1:2 to 3:2 caused a change in the morphology, to more dendritic in nature, whereas a higher content of Bi produced a smoother surface. The Sn/Bi ratio of 35:65 wt % found in the alloy deposited at −0.4 V from the 2:1 mixture could almost be reversed by making the deposition potential more negative (−0.6 V). The X‐ray diffraction patterns of the individual metals and the alloys revealed crystalline Sn and Bi in addition to AuSn and AuSn_2_ intermetallic phases formed between the gold substrates and the Sn‐containing deposits.

## Experimental Section

### Chemicals and Electrolyte Preparation

[N*n*Bu_4_][SnCl_3_] and [N*n*Bu_4_][BiCl_4_] were prepared as described in the literature.^4^, ^5^ The salts were added to 1:2 molar ratio mixtures of choline chloride/ethylene glycol (12CE) prepared as previously described.^39^ The solutions with a concentration of 10 mmol L^−1^ were heated to 60 °C and stirred until the salt had completely dissolved. Whereas [N*n*Bu_4_][SnCl_3_] dissolved in a few minutes, [N*n*Bu_4_][BiCl_4_] was considerably less soluble, and therefore, this mixture was stirred overnight.

### Electrochemistry

The electrochemical experiments were performed in a three‐electrode cell within a dry, argon‐filled glove box (MBraun 150‐B‐G‐II, <1 ppm H_2_O/O_2_). All experiments were conducted at the glove box temperature (30 °C). A silver wire was used as a quasireference electrode (RE). The RE was kept in a compartment containing 12CE, separated by a microporous frit, and placed approximately 3 mm from the working electrode (WE). Platinum wires were used as inert counter electrodes (CEs). Stationary (SE) and rotating disc (RDE) electrodes were used as WEs. The electrodes were made of nonporous glassy carbon (GC, SE 0.071 cm^2^, RDE 0.126 cm^2^), platinum (SE 0.071 cm^2^, RDE 0.126 cm^2^), and gold (0.071 cm^2^). All WEs were polished to a mirror finish with alumina powder (0.03 μm grain size), rinsed with deionized water and acetone, and dried in air at 60 °C. Sn, Bi, and Sn–Bi films were electrodeposited potentiostatically onto evaporated gold‐on‐glass slides (1 cm^2^) that consisted of microscope slides with a 5 nm chromium adhesion layer and 100 nm of gold. Prior to electrochemical experiments, the gold‐on‐glass slides were cleaned by ultrasonic agitation in 2‐propanol for 10 min and then dried under a flow of nitrogen. A Metrohm Echo Chemie Autolab PGSTAT100 potentiostat/galvanostat controlled by NOVA 1.10 software and connected to the glove box was used to perform cyclic voltammetry and potential step techniques under computer control.

### Materials Characterization

The surface morphology was analyzed with a scanning electron microscope (ESEM Tescan 500 PA) equipped with an energy‐dispersive X‐ray (EDX) analyzer (INCA x‐act Oxford Instruments) for determining the elemental composition. X‐ray diffraction (XRD) was performed with an X‐ray powder diffractometer (Bruker D8 advance, CuKα wavelength of 1.5406 Å). Raman spectra of the Bi^III^ solutions were recorded with a Horiba Jobin Yvon confocal Raman microscope with a *λ*=633 nm focused laser beam. Due to the strong fluorescence of [SnCl_3_]^−^,^61^ these measurements were performed in a PerkinElmer Raman Station 400F with a focused *λ*=785 nm (NIR) laser. Quartz cuvettes with a path length of 2 mm were filled with electrolyte (1 mL) in the glove box, closed with a Teflon cap, and sealed with Parafilm. Five 30 s scans were recorded for each sample. ^119^Sn{^1^H} NMR spectra were recorded from a 10 mm solution of [N*n*Bu_4_][SnCl_3_] in 12CE containing a D_2_O capillary as lock and TbCl_3_ as a relaxation agent. Data acquisition used a Bruker AVII400 spectrometer and chemical shifts are referenced to SnMe_4_ (*δ*=0 ppm).

## Conflict of interest

*The authors declare no conflict of interest*.

## Supporting information

As a service to our authors and readers, this journal provides supporting information supplied by the authors. Such materials are peer reviewed and may be re‐organized for online delivery, but are not copy‐edited or typeset. Technical support issues arising from supporting information (other than missing files) should be addressed to the authors.

Supplementary
