# Supplementary material for: Tin, Bismuth, and Tin–Bismuth Alloy Electrodeposition from Chlorometalate Salts in Deep Eutectic Solvents
Source: ChemistryOpen. 2017 Apr 13;6(3):393–401. doi: 10.1002/open.201700045 (PMC5474671; doi:10.1002/open.201700045)
Supplement: Supplementary file 1 — Supplementary [file OPEN-6-393-s001.pdf]

ChemistryOPEN

Supporting Information

© 2017 The Authors. Published by Wiley-VCH Verlag GmbH & Co. KGaA, Weinheim

Tin, Bismuth, and Tin–Bismuth Alloy Electrodeposition from Chlorometalate Salts in Deep Eutectic Solvents

Luciana Vieira,[a] Jennifer Burt,[b] Peter W. Richardson,[b] Daniel Schloffer,[a] David Fuchs,[a] Alwin Moser,[a] Philip N. Bartlett,[b] Gillian Reid,[b] and Bernhard Gollas*[a]

open_201700045_sm_miscellaneous_information.pdf

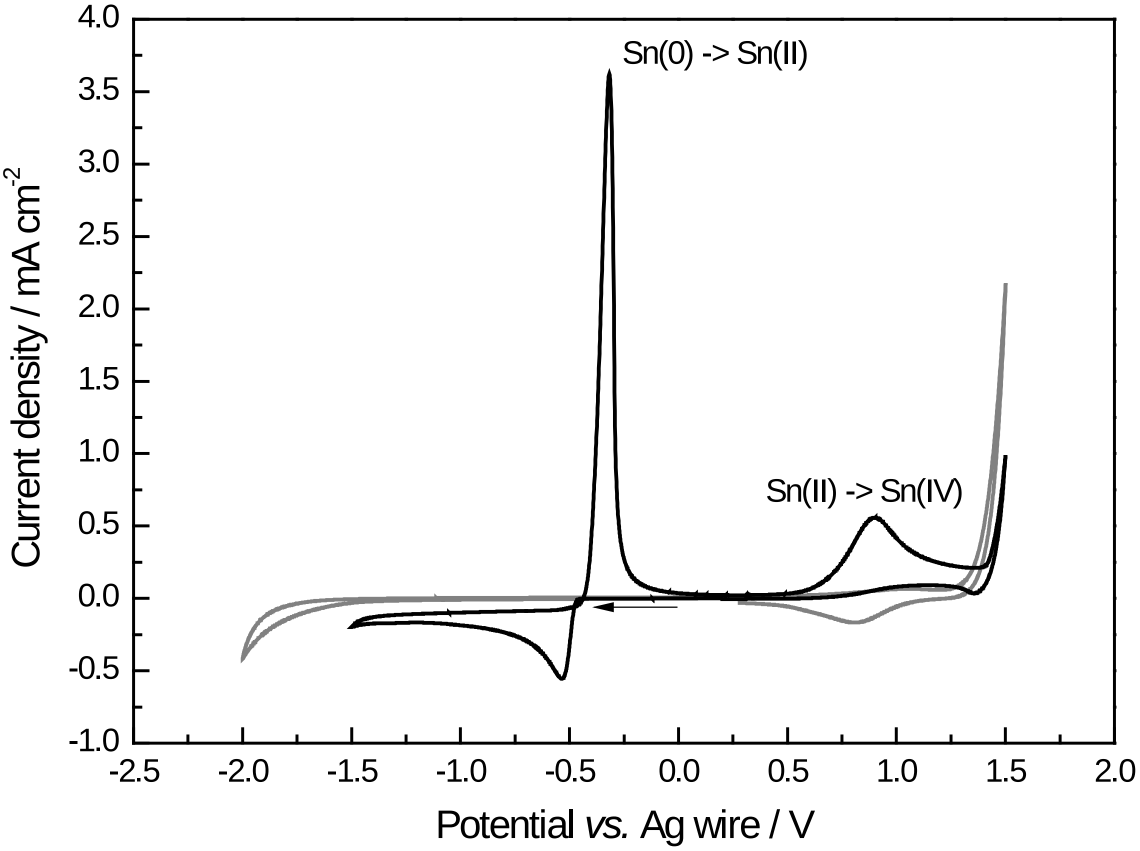

Figure SI 1: CV of 10 mM [N^nBu_4][$SnCl_3$] in 12CE on a GC disk electrode (0.07 cm^2) at v = 50 mV s^{-1} and room temperature. The sweep started at open circuit potential with a negative sweep direction. The light gray scan is the background CV (pure 12CE) with the same scan rate and temperature. The CV of 10 mM [N^nBu_4][$SnCl_3$] in 12CE shows the chemically irreversible oxidation of Sn(II) to Sn(IV) at +0.9 V.

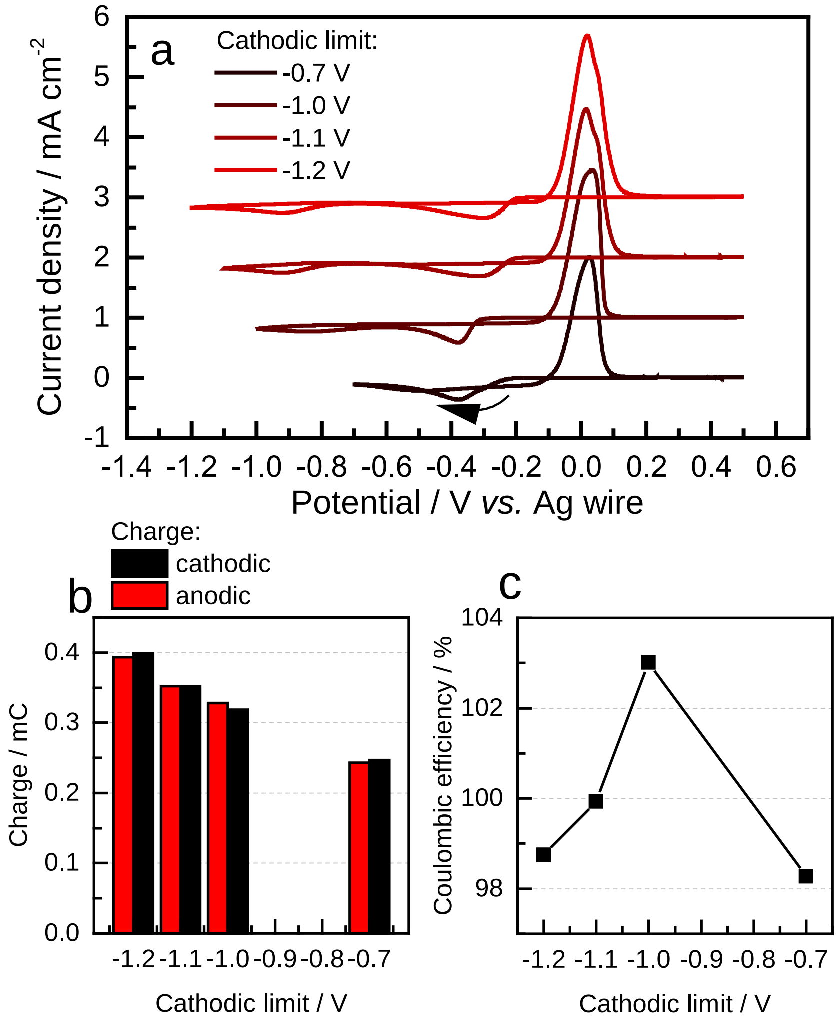

Figure SI 2: a) CV of 10 mM [N^nBu_4][$BiCl_4$] in 12CE on a GC disk electrode (0.07 cm^2) at v = 50 mV s^{-1} and room temperature with different cathodic switching potentials. The sweeps started at open circuit potential with a negative sweep direction, b) cathodic and anodic total charge passed during the CV, and c) coulombic efficiencies.

a)

Element	Weight %	Atomic %	Sn:Bi wt% ratio
C K	18.43	72.19	
N K	-1.74	-5.86	
O K	4.55	13.39	
Sn L	11.08	4.39	34.7
Au M	46.87	11.20	
Bi M	20.82	4.69	65.2
Total	100.00		100

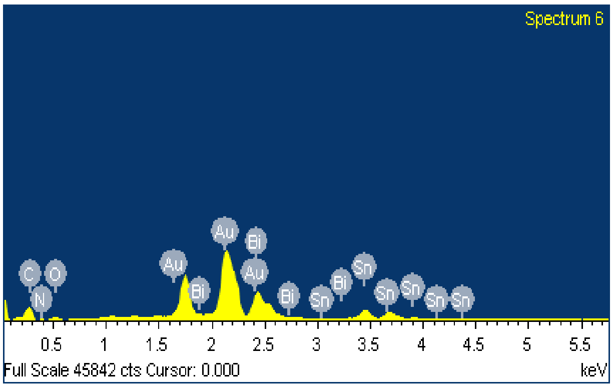

b)

Elemen t	Weight %	Atomic %	Sn:Bi wt% ratio
C K	5.95	46.81	
Sn L	27.70	22.04	53.5
Au M	42.27	20.27	
Bi M	24.07	10.88	46.5
Totals	100.00		100

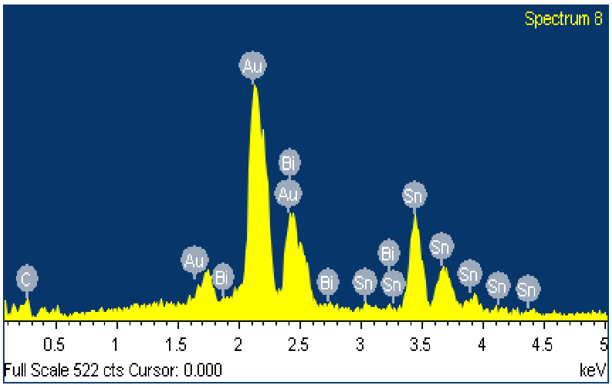

c)

Elemen t	Weight %	Atomic %	Sn:Bi wt% ratio
O K	8.17	48.07	
Sn L	27.12	21.51	59.2
Au M	45.97	21.97	
Bi M	18.74	8.44	40.8
Totals	100.00		

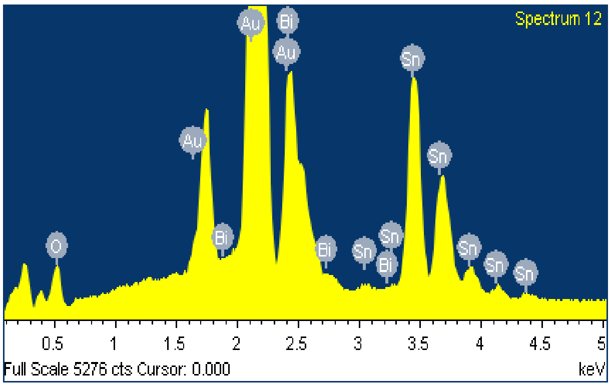

Figure SI 3: EDX spectra and elemental quantification of the Sn-Bi alloys deposited at potentials of -0.4 V a), -0.5 V b), and -0.6 V c).

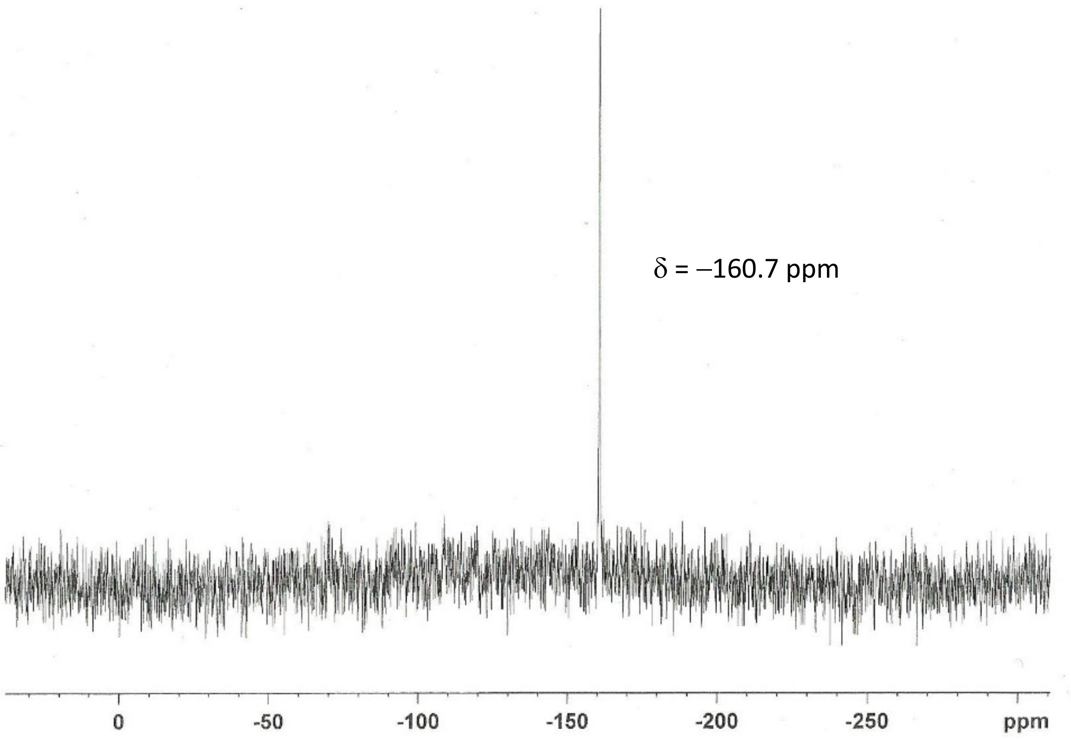

Figure SI 4: $^{119}Sn\{^1H\}$ NMR spectrum of $[N^nBu_4][SnCl_3]$ in 12CE (relaxation agent = $TbCl_3$).

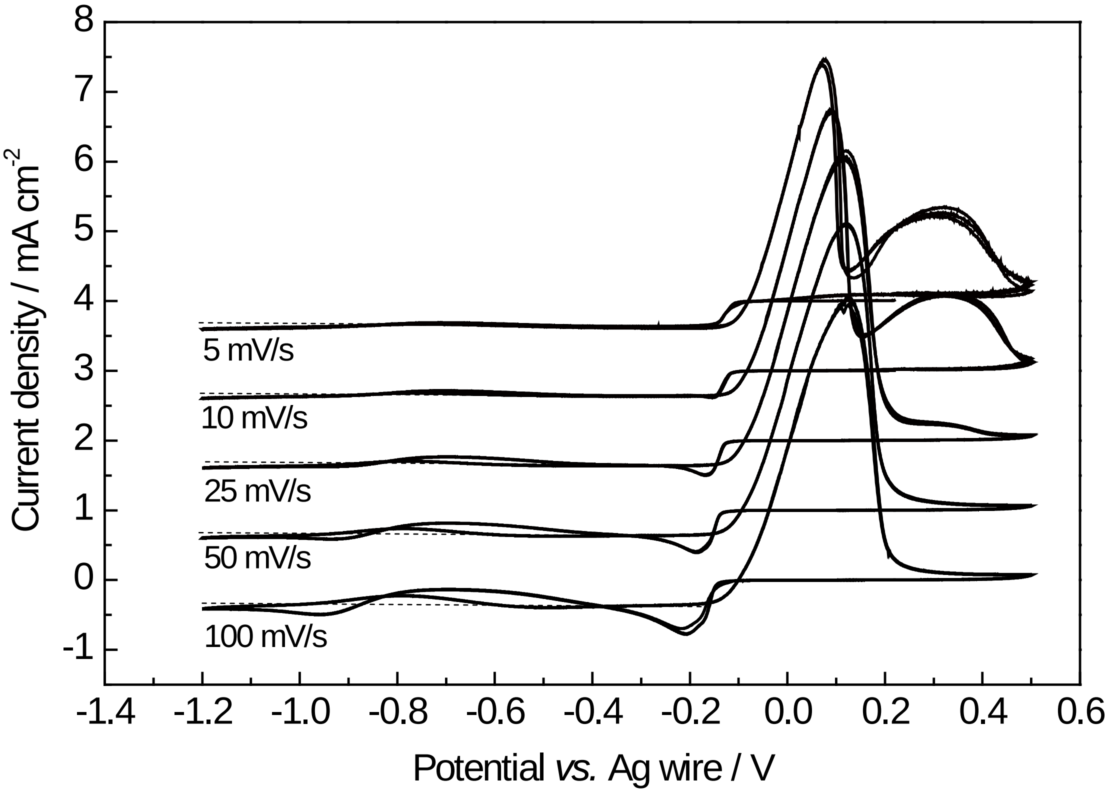

Figure SI 5: RDE CVs of 10 mmol·L^{-1} $[N^nBu_4][BiCl_4]$ in 12CE on a GC disk electrode (0.126 cm^2) at 1000 rpm starting from OCP at +0.22 V with a negative sweep direction, T = 30°C. The dotted lines are extrapolated steady current plateaux of the first reduction and intended as guide to the eye for a better recognition of the second reduction wave.

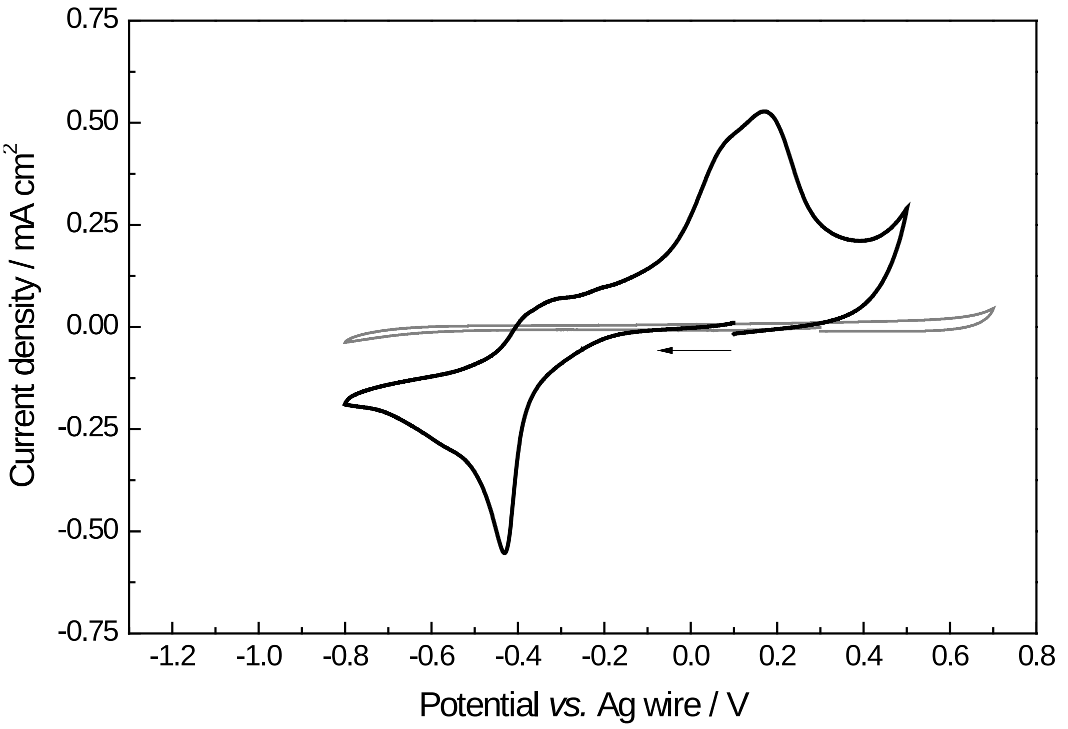

Figure SI 6: CV of 10 mM [N^{n}Bu$_4$][SnCl$_3$] in 12CE on a Au disk electrode (0.07 cm^2) at v = 50 mV s^{-1} and room temperature. The sweep started at open circuit potential with a negative sweep direction. The light gray scan is the background CV (pure 12CE) with the same scan rate and temperature.

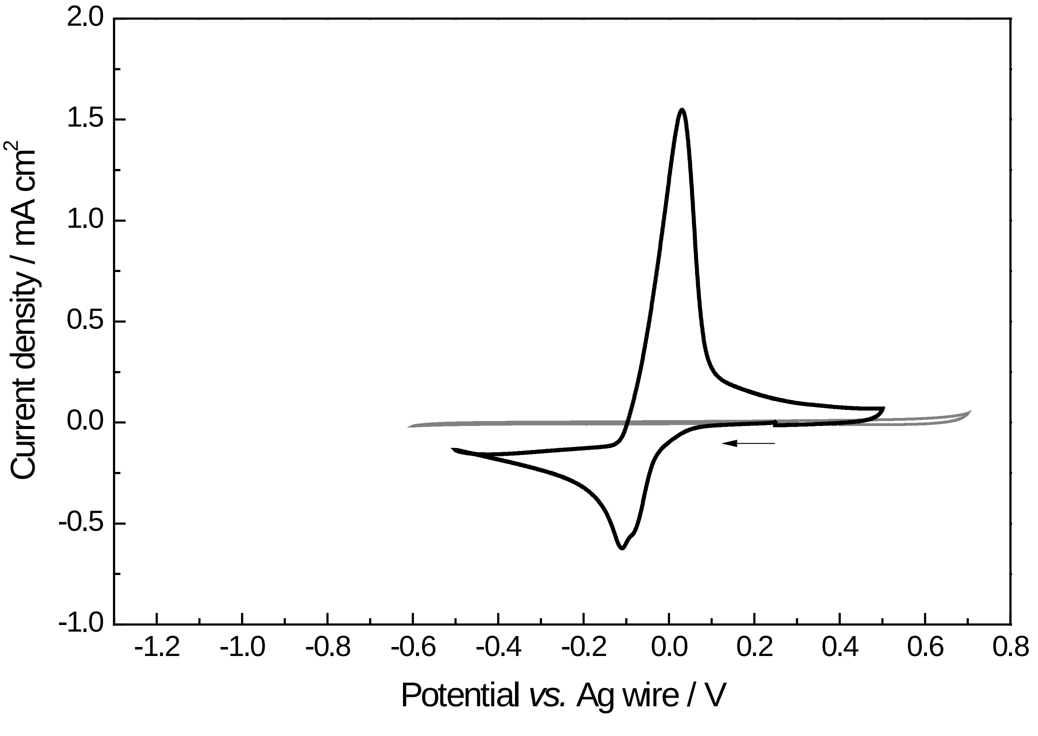

Figure SI 7: CV of 10 mM [N^{n}Bu$_4$][BiCl$_4$] in 12CE on a Au disk electrode (0.07 cm^2) at v = 50 mV s^{-1} and room temperature. The sweep started at open circuit potential with a negative sweep direction. The light gray scan is the background CV (pure 12CE) at the same scan rate and temperature.

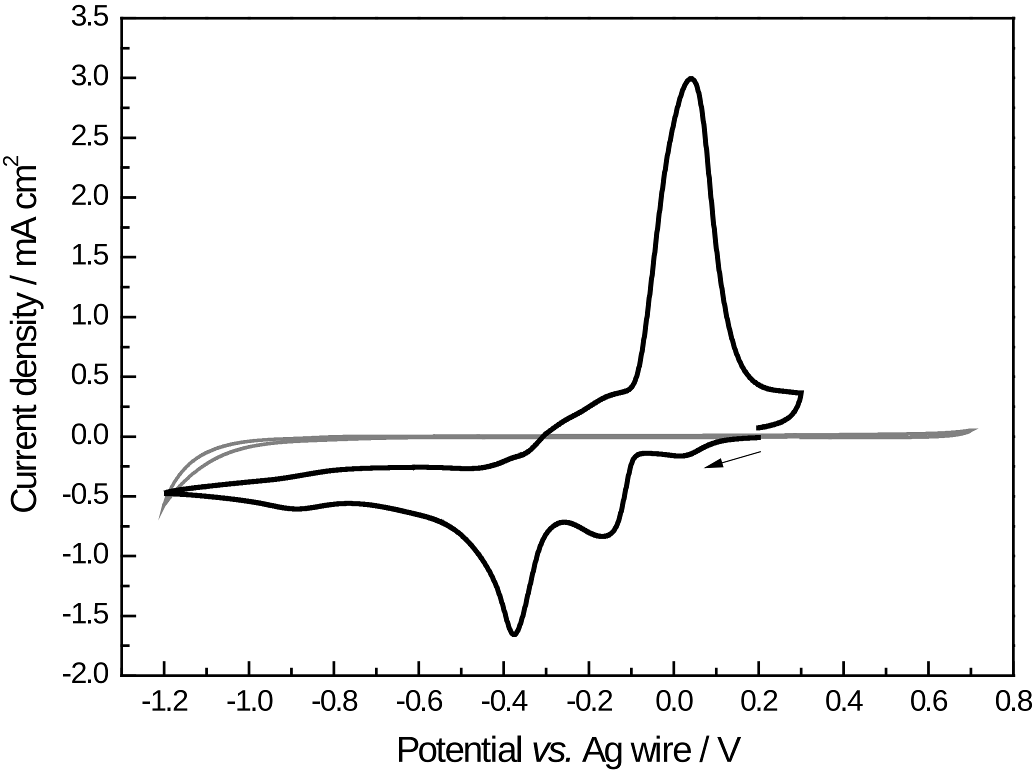

Figure SI 8: CV of 12CE containing $[NBu_4][SnCl_3]/[NBu_4][BiCl_4]$ of a molar ratio of 2:1 (20/10 mM) on a Au disk electrode (0.07 cm^2) at v = 50 mV s^{-1} and room temperature. The sweep started at open circuit potential with a negative sweep direction. The light gray scan is the background CV (pure 12CE) at the same scan rate and temperature.
